# The relationship between post-traumatic stress disorder and suicidal ideation among shidu parents: the role of stigma and social support

**DOI:** 10.1186/s12888-019-2353-7

**Published:** 2019-11-08

**Authors:** Qiong Wang, Longfei Ren, Wenhao Wang, Weihua Xu, Yang Wang

**Affiliations:** 1 Department of Social Medicine, School of Public Health, China Medical University, No. 77 Puhe Road, Shenyang North New District, Shenyang, Liaoning 110122 People’s Republic of China; 2 Department of Medical Care and Maintenance, Health Commission of Shenyang, No. 13 Beiqi Road, Heping District, Shenyang, Liaoning 110003 People’s Republic of China

**Keywords:** Suicidal ideation, PTSD, Stigma, Social support, Shidu parents

## Abstract

**Background:**

Losing an only child is a particularly traumatic and heartbreaking event for parents, which can trigger a lot of emotional responses, including PTSD and suicidal ideation (SI). The objectives of this study were mainly to identify predictors of SI and examine the interactions of PTSD with stigma and social support on SI among shidu parents.

**Methods:**

A total of 507 shidu parents from Shenyang, China were included in this cross-sectional study. Bivariate logistic regression analyses were conducted to explore risk or protective factors associated with SI. Interactions of PTSD with stigma and social support on SI were also examined by bivariate logistic regression analyses.

**Results:**

The prevalence of SI among shidu parents was 11.24%. PTSD (OR = 2.23, *p* < 0.05) and stigma (OR = 4.66, *p* < 0.01) were positively associated with SI. Social support was negatively associated with SI (OR = 0.90, *p* < 0.01). For individuals with PTSD, the presence of stigma was more likely to lead to SI. For individuals with PTSD, an increased level of social support was less likely to lead to SI.

**Conclusions:**

SI is a serious issue among shidu parents. Stigma aggravated the effect of PTSD on SI, while social support buffered the effect of PTSD on SI among shidu parents.

## Background

Parents who lose their only child and can not conceive another child are known as shidu parents in China. It is estimated that there are about 76,000 new shidu families occurring each year [1]. As a large aging group, shidu parents are suffering from physical and mental illnesses caused by traumatic loss and the unique social and cultural context in China [2]. As Chinese families are highly child-centered and children are the main source of care-giving and mental dependence for parents, losing the only child means that the love, money, and time that parents have invested are gone, which can extremely increase the financial and social burden for shidu parents [3]. In addition, the important tradition of passing down the family name is a big part of the parents’ responsibility and obligation in Confucianism, the death of the only child means the termination of the family name and is a sign of bad luck, which may bring feelings of stigma to shidu parents [2]. Some studies have examined the influence of losing the only child on parental mental problems, such as post-traumatic stress disorder (PTSD), loneliness, depression and anxiety [3–6]. However, suicide, which is often regarded as a direct consequence of mental disorders [7], is not well studied among Chinese shidu parents. As a major health problem worldwide, suicide has been found to be one of the primary health causes of mortality and morbidity [8]. In China, the suicide rate was 7.8 per 100,000 people in 2012, which ranks second globally [9]. Traumatic experiences (i.e. disaster, violence, abuse, and loss) have been identified to increase the risk of suicide [10, 11]. Losing a child is a particularly traumatic and heartbreaking event for parents, which can trigger lots of emotional responses, such as increased risk of suicide, PTSD and depression [12, 13]. Suicidal ideation (SI) is an important predictor and inevitable stage for suicide behavior [14, 15]. Previous studies have reported high prevalence of SI among bereaved parents. Ariapooran et al. found that 46.6% of the bereaved women had some degree of SI [16]. Lee et al. found that bereaved parents were more likely to have SI than non-bereaved parents [17]. As an unique bereaved group, Chinese shidu parents may be more likely to generate SI. Therefore, exploring the prevalence of SI and its associated risk and protective factors is critical for the prevention of suicidal behavior among Chinese shidu parents.

Sociodemographic factors of SI have also been explored in previous studies among different populations. A study found that gender, age, education, marital status, household economic status were the major sociodemographic factors associated with SI among earthquake adult survivors [10]. Ran et al. found that gender and loss of family members had strong associations with SI among adolescent survivors 18 months after the earthquake [18]. Additionally, some studies have explored the relationship between sociodemographic characteristics and SI among seniors in China [19–21]. However, what do the sociodemographic characteristics of Chinese shidu parents contribute to their SI has not been examined.

PTSD is the most common mental disorder among individuals with a history of traumatic experiences [5, 10]. According to the Diagnostic and Statistical Manual of Mental Disorders, Fifth Edition (DSM-5), PTSD is characterized as one of the new classes of “trauma and stressor-related disorders”, including intrusion symptom, avoidance symptom, cognition and mood symptoms, and arousal symptoms [22]. A high prevalence of PTSD among shidu parents has been found in previous studies [4, 23]. Yin et al. indicated that shidu parents obtained higher PTSD scores than parents who did not lose a child [4]. Xu et al. found that the prevalence of PTSD in mothers who lost a child was twice as high as that of mothers who had a subsequent child after the 2008 Sichuan earthquake [23]. Furthermore, research revealed that individuals with PTSD were more likely to endorse SI than those without PTSD [24, 25]. Moreover, people who had PTSD symptoms and did not recover for a long time may have a greater chance of suicide [26]. Although the relationship between PTSD and SI has been examined in previous studies [27, 28], limited studies have examined the relationship between PTSD and SI as well as the potentially aggravating or buffering pathways between PTSD and SI among Chinese shidu parents.

From a risk perspective, stigma may play an important role for occurrence of SI among shidu parents. Previous studies have found feelings of stigma were common among bereaved parents [29, 30]. In a cross-sectional study conducted in UK, high levels of perceived stigma have been found among people bereaved by suicide and other sudden deaths [31]. Stigma has been reported as having a significantly positive association with SI among populations at risk of psychosis in a longitudinal study [32]. Also, stigma has been found to be one of the risk factors for SI among adolescents living with HIV [33]. However, no studies were conducted specifically to examine the relationship between stigma and SI among shidu parents. In addition, stigma was reported to be the biggest barrier to help-seeking [34, 35]. A number of studies indicated that individuals who suffered from PTSD were less likely to seek help [34, 36] and low rates of help-seeking were associated with higher suicide rates [7]. Moreover, in a randomized controlled trial, after reducing feelings of stigma through online stigma reduction intervention, refugees with PTSD reported greater help-seeking behaviors [37]. Thus, we hypothesized that the presence of stigma may aggravate the effect of PTSD on SI.

From a protective perspective, social support is one of the most discussed factors likely to decrease SI. Previous studies provided initial evidence that social support can decrease SI. Ariapooran et al. found that higher social support can lead to a reduction in SI in bereaved women who have experienced the death of a young person [16]. Endo et al. identified a strongly inverse relationship between perceived social support and the severity of SI [38]. However, to our knowledge, no studies were conducted to examine the relationship between social support and SI among shidu parents. Additionally, high levels of social support have been linked with positive outcomes or a lower risk of suicide in people with PTSD [39]. Panagioti et al. found that perceived social support can buffer the impact of PTSD on suicidal behaviors [39]. Casale et al. found that social support buffered the negative effects of stigma on SI among adolescents living with HIV [33]. In a study of shidu parents from the 2008 Sichuan earthquake, Wang and Xu found that social support had an important impact on moderating the relationship between PTSD and quality of life [5]. However, whether or not social support can buffer the effect of PTSD on SI among shidu parents has not been examined.

Based on the situation described above, the aims of this study were 1) to explore the prevalence of SI and its associated sociodemographic factors among shidu parents, 2) to examine the relationships among PTSD, stigma, social support and SI among shidu parents, 3) to examine the interaction effects of PTSD and stigma on SI and the interaction effect of PTSD and social support on SI among shidu parents. We hypothesized that 1) PTSD and stigma are positively associated with SI among shidu parents. 2) social support is negatively associated with SI among shidu parents. 3) stigma aggravates the effect of PTSD on SI among shidu parents 4) social support buffers the effect of PTSD on SI among shidu parents.

## Methods

### Participants

A cross-sectional study was conducted in Shenyang, China from March 2017 to September 2017. Participants were recruited from five urban districts of Shenyang, Liaoning Province located in the Northeast of China. In each district, 15 communities were randomly selected. In each community, we invited all shidu parents who satisfied the inclusion criteria to participate this study. Participants participated in the study voluntarily and anonymously and their information were kept strictly confidential and only used for this research. Of the 595 enrolled parents, 79 refused to participate, and 9 of the rest 516 were excluded from the analysis because missing data was over 30%. Finally, a total number of 507 shidu parents were included in the analysis, and the effective response rate is 85.21%. There were no significant differences for sociodemographic characteristics of parents who agreed and who refused to participate this survey.

This study received ethical approval from the Committee on Human Experimentation of China Medical University and all research processes met ethical standards.

### Participant recruitment

Community family planning commissioners in different communities gave great help to this survey, because they communicated and visited with shidu parents periodically in their daily lives and kept close relationships with shidu parents. Firstly, before the survey, a 2-day training session was conducted to all interviewers and community family planning commissioners. Secondly, inclusion criteria of enrolled parents included: (1) were living in the community for over a half year; (2) lost their only child and have not given birth or adopted another child; (3) were able to communicate clearly [40]. Parents who suffered from mental dementia, retardation or several mental disorders were excluded [40]. Thirdly, the commissioners called parents who satisfied the inclusion criteria to tell them the purposes of this study and further invite them to complete self-reported questionnaires in the office of community. Fourthly, we explained to every participant the the purposes and significance of the survey before obtaining their written informed contents. The commissioners would visit their homes to complete the questionnaires on condition that parents felt uncomfortable coming to the office. Answers of the questionnaire were acquired by face-to-face interviews when participants had difficulties to self-report. Finally, after completing the survey, each participant would get an honorarium of 100 RMB.

### Sociodemographic characteristics and loss-related information

Sociodemographic characteristics included gender, age, marital status, education, annual household income, marital satisfaction and chronic disease. If any of the chronic diseases (eg, hypertension, diabetes, cardiovascular disease, chronic hepatitis, ulcer, gout and arthritis) had ever been diagnosed, chronic disease was defined as ‘yes’. The loss-related information included gender of the child, age of the child at death, reason of the child’s death, time since the child’s death.

### Suicidal ideation

Suicidal ideation was assessed by using a question which has been used in the US National Comorbidity Survey(NCS) [15]. The participants were asked about the SI, “Have you ever seriously thought about committing suicide since losing your only child?” Answer was coded as ‘yes’(1) or ‘no’(0). This question was widely used to assess SI and has been found to have a good validity [10, 20].

### Post-traumatic stress disorder

We used the PTSD Checklist for DSM-5 (PCL-5), a 20-item self-report measure defined in the DSM-5 to assess the PTSD symptoms [22]. Each item is rated on a five-point rating scale from 0 (not at all) to 4 (extremely) to reflect the severity of a particular symptom during the past month. Summed scores indicate greater PTSD symptom severity (range: 0–80). A total cutoff score of ≥33 was used to identify PTSD symptoms [41]. The Chinese version of the PCL-5 has been validated and widely used in Chinese population [42, 43]. Cronbach’s alpha coefficient of the scale in this study was 0.965.

### Social support

Social support was assessed by using the Duke-UNC Functional Social Support Questionnaire (FSSQ), which is a 8-item questionnaire [44]. Each item is rated on a five-point Likert scale ranging from 1 (much less than I would like) to 5 (as much as I would like). Participants with higher scores had greater social support. The Chinese version of the FSSQ has shown good reliability and validity [45]. In the present study, Cronbach’s alpha coefficient of the scale was 0.938. Since there is no recommended cutoff point for FSSQ, the median of the total scores in this study was used as a cutoff point to stratify the sample into high versus low levels of social support in the interaction effect analyses [10].

### Stigma

Stigma was assessed by the question, “have you ever felt that you had been stigmatized or discriminated by others because of the death of your child?” Answer was coded by ‘yes’ into ‘1’ and ‘no’ into ‘0’.

### Statistical analysis

The SPSS20.0 was used for all data analysis. Statistical significance was set as a two-tailed *p*-value < 0.05. The sample mean was used to replace the missing values of continuous variables. First, the prevalence of SI among different sociodemographic groups were compared using Chi-square(χ2) or independent *t*-tests. Second, bivariate logistic regression analyses were performed to determine the association between SI and the sociodemographic variables, PTSD, stigma and social support. Finally, according to the presence or absence of PTSD and stigma, the whole sample was stratified into 4 groups to examine the interaction effects of PTSD and stigma on SI. Meanwhile, the whole sample was also stratified into 4 groups on the basis of the level of PTSD and social support to examine the interaction effects between PTSD and social support on SI.

## Results

### Prevalence of SI among shidu parents

Sociodemographic variables and the results of Chi-square or independent *t*-tests were shown in Table 1. Among the 507 respondents, approximately 11.24% were reported as having SI. Prevalence of SI in females was twice as that in males. There were statistical significant differences among the variables of marital status and chronic disease. Within annual household income groups, the respondents in the poor group had the highest prevalence of SI. The participants who were unsatisfied with marriage had a higher prevalence of SI than those who were satisfied. The participants with PTSD were more likely to endorse SI compared to those without PTSD. The respondents with stigma had a remarkably higher prevalence of SI than those without stigma. For parents with or without SI, the mean scores of social support were 16.84 (SD = 6.70) and 24.55 (SD = 7.32), respectively (*p* < 0.01*)*.

**Table 1:** Descriptive characteristics associated with SI among shidu parents

Characteristics	N (%)	Suicidal ideation			
		No (%)	Yes (%)	χ2/t	*p*-value
Gender				5.46	0.022
male	206(40.63)	191(42.44)	15(26.32)		
female	301(59.37)	259(57.56)	42(73.68)		
Age				2.94	0.23
< 60	194(39.19)	168(37.33)	26(45.61)		
60~70	269(51.92)	240(53.3)	29(50.88)		
≥ 70	44(8.89)	42(9.33)	2(3.51)		
Marital status				7.22	0.009
Couple	322(63.51)	295(65.56)	27(47.37)		
Single	185(36.49)	155(34.44)	30(52.63)		
Education				0.05	0.976
Middle school or under	336(66.27)	298(66.22)	38(66.67)		
Senior high school	141(27.81)	125(27.78)	16(28.07)		
Undergraduate or above	30(5.92)	27(6.00)	3(5.26)		
Chronic disease				8.67	0.004
No	226(44.58)	211(46.89)	15(26.32)		
Yes	281(55.42)	239(53.11)	42(73.68)		
Annual household income				33.67	< 0.001
> 30,000	236(46.55)	220(48.89)	16(28.07)		
10,000–30,000	232(45.76)	206(45.78)	26(45.61)		
< 10,000	39(7.69)	24(5.33)	15(26.32)		
Marital satisfaction				14.04	0.001
Satisfactory	413(81.46)	378(85.14)	35(64.81)		
Unsatisfactory	85(16.76)	66(14.86)	19(35.19)		
Missing	9(1.78)				
Gender of lost child				0.09	0.770
Male	329(64.89)	293(65.11)	36(63.16)		
Female	178(35.11)	157(34.89)	21(36.84)		
Time since the child’s death				3.62	0.164
< 5	203(40.04)	176(39.64)	27(47.37)		
6–15	180(35.50)	166(37.39)	14(24.56)		
> 15	118(23.27)	102(22.97)	16(28.07)		
Missing	6(1.18)				
Age of the child at death				0.60	0.441
< 16	79(15.58)	68(15.32)	11(19.30)		
≥ 16	422(83.24)	376(84.68)	46(80.70)		
Missing	6(1.18)				
Reason of the child’s death				1.58	0.455
Accident	134(26.43)	116(25.95)	18(32.14)		
Chronic or acute diseases	349(68.84)	312(69.80)	37(66.07)		
Mental illness or suicide	20(3.94)	19(4.25)	1(1.79)		
Missing	4(0.79)				
PTSD				61.93	< 0.001
No	383(75.54)	364(80.89)	19(33.33)		
Yes	124(24.46)	86(19.11)	38(66.67)		
Stigma				59.05	< 0.001
No	362(71.40)	346(76.89)	16(28.07)		
Yes	145(28.60)	104(23.11)	41(71.93)		
Social support ^a^	507	24.55(7.32)	16.84(6.70)	7.56	< 0.001

^a^ Social support was presented as Mean ± SD, and analyzed by using t-test

### Associations among sociodemographic factors, PTSD, stigma, social support and SI

Table 2 displayed the results of the bivariate logistic regression model. Sociodemographic factors of SI among shidu parents were gender (OR = 2.81, *p* < 0.05), annual household income (OR = 4.67, *p* < 0.01). SI was positively associated with PTSD (OR = 2.23, *p* < 0.05) and stigma (OR = 4.66, *p* < 0.01), while SI was negatively associated with social support (OR = 0.90, *p* < 0.01) .

**Table 2:** Associations among sociodemographic factors, PTSD, stigma, social support and SI in shidu parents

variables	Model 1	
	OR	95%CI
Gender		
male	1.00	
female	2.81^*^	1.27–6.22
Marital status		
Couple	1.00	
Single	1.10	0.48–2.48
Chronic disease		
No	1.00	
Yes	1.60	0.76–3.35
Annual household income		
> 30,000	1.00^**^	
10,000–30,000	0.92	0.40–2.10
< 10,000	4.67^**^	1.53–14.30
Marital satisfaction		
Satisfactory	1.00	
Unsatisfactory	1.60	0.69–3.74
PTSD		
No	1.00	
Yes	2.23^*^	1.05–4.74
Stigma		
No	1.00	
Yes	4.66^**^	2.18–9.98
Social support	0.90^**^	0.86–0.95

*p < 0.05, ** *p* < 0.01

### Interactions of PTSD with stigma and social support on SI

Table 3 showed the interaction effects of PTSD with stigma and social support on SI after controlling for the variables of gender and annual household income. Respondents with PTSD and stigma were more likely to report SI (OR = 22.18, 95% CI = 9.35–52.61) compared to those without PTSD and stigma. Respondents with PTSD and low level of social support were more likely to report SI (OR = 16.72, 95% CI = 6.82–40.95) compared to those without PTSD and received high level of social support.

**Table 3:** Interactions of PTSD with stigma and social support on SI

Model 2	OR	95%CI	Model 3	OR	95%CI
None PTSD * none stigma	1		None PTSD * high social support	1	
None PTSD * stigma	7.79^**^	2.94–20.66	None PTSD * low social support	3.83^**^	1.45–10.12
PTSD * none stigma	4.19^*^	1.35–12.95	PTSD * high social support	5.75^**^	1.78–18.60
PTSD * stigma	22.18^**^	9.35–52.61	PTSD * low social support	16.72^**^	6.82–40.95

*p < 0.05, ** *p* < 0.01

Note: Gender and annual household income were controlled in these analyses

## Discussion

In this study, the prevalence of SI among shidu parents was 11.24%, which was much higher than that among the seniors in Shandong, China (4.2%) [20] and among the Wenchuan earthquake survivors (9.06%) [10]. The prevalence of this study was similar with the reported SI rate of bereaved parents reported by Murphy [46]. Murphy et al. observed 175 parents bereaved by the violent deaths of their children for 5 yrs, and found that 9% of them had SI in initial 4 months and 13% of them had SI after 5 yrs [46]. Such findings across studies may well highlight the imperative need to develop intervention for shidu parents to prevent suicide. Our study examined the relationship between SI and sociodemographic variables, such as gender, age, education, annual household income and so on. Results of logistic regression showed that SI was more prevalent in those who were female and lived with lower annual household income, which is consistent with previous research [10, 20, 21]. The gender differences in SI may be attributed to social factors such as the gender role and position of women in mainland China [21]. In China, mothers spend more time raising their only child and have a greater emotional dependence on their families than fathers [4, 17], therefore, mothers may have a higher likelihood of developing SI after losing their only child who acted as the link of a family. For shidu parents, losing their only child means losing the main economic support in context of Chinese tradition [47], which may cause negative thoughts, including SI. Additionally, Lee et al. indicated that mothers were more likely to suffer substantial financial stress than fathers after the death of a child [17]. These findings indicate that the prevention for shidu parents need to focus more on females and financial support should be provided for shidu parents with economic difficulties.

Consistent with our hypothesis 1, this study found that PTSD was positively associated to the presence of SI. Individuals who experienced PTSD were significantly more likely to report current SI compared to those who did not report PTSD. This result corresponded with previous studies which indicated that PTSD had a powerful effect on SI [10, 27]. The factors of gender and economic status have been found to be the main sociodemographic factors affecting PTSD among shidu parents [48]. In addition, a previous study found that PTSD was negatively associated with quality of life among shidu parents [5]. These finding implied a urge to pay more attention to develop interventions targeting shidu parents with PTSD, so as to decrease their SI and other negative consequences caused by PTSD.

Our results showed that stigma was positively associated with SI, which was consistent with our hypothesis 1 and previous studies [32, 33]. Zheng and Lawson indicated that the first unique factor having an impact on the mental health of shidu parents was the cultural stigmatization related to a child’s death [49]. Chinese culture views the death of children as a sign of bad luck and a taboo topic associated with numerous superstitions and customs [49]. Furthermore, the death of the only child makes shidu parents perceive stigma because they are unable to pass down the family name through their biological offspring causing them to think that they are unfilial and shameful to their ancestors [3]. Therefore, understanding negative consequences of stigma on SI and alleviating the stigma among shidu parents are of high priority in further studies.

Consistent with our hypothesis 2, our findings also showed that lower level of social support was significantly associated with an increased risk of SI. This result was consistent with a study which indicated that people who suffered from SI reported low social support [38]. Bereavement means the loss of social and financial support, and that further leads to negative thoughts, including SI [16]. Research has found that bereaved parents perceived lower social support from friends or family than they expected [50]. Moreover, Wei et al. found that shidu parents had less support in terms of practical support, emotional support, social interactions, and thinning social support networks [47]. Thus, intervention increasing social support should be conducted to shidu parents. In addition, Cao et al. found that social support partially mediated the relationship between social capital and depression and indicated that social capital conferred social support to individuals to prevent the development of mental disorders [51]. Wu et al. suggested that higher family and community social capital was associated with lower level of mental disorders [52]. Thus, the implications of social capital including family and community capital should also be considered when developing interventions to prevent SI of shidu parents.

Our results fully support our hypothesis 3 that stigma significantly aggravated the effect of PTSD on SI. For individuals with PTSD, their SI did increase significantly when they perceived stigma. This finding was similar with the results of Farrelly et al. that for 194 individuals diagnosed with depression and other disorders, the stigma they perceived contributed to making a suicide attempt [53]. Previous studies also found that most people with mental illness and a history of SI felt that stigma may deepen their negative motions [54]. Thus, based on the present data, reducing stigma among individuals with PTSD can reduce the SI. Future studies should consider the importance of targeting stigma in the development of preventive interventions addressing SI for shidu parents with PTSD. Our finding demonstrated that the negative effects of PTSD on SI were alleviated by social support to some extent, which corresponded with our hypothesis 4 and the results of some similar studies [5, 33]. Panagioti et al. indicated that for individuals that reported high social support, their levels of suicidal behavior did not increase significantly even when they suffered from PTSD [39]. Specific risk factors tend to stimulate suicidal behaviors while certain protective factors help to insulate individuals against these stimulants [55]. Similarly, PTSD has negative effects on SI while social support can impede the negative effects and further prevent the occurrence of SI. Thus, it is also necessary to consider the buffer role of social support in the development of preventive interventions addressing SI for shidu parents with PTSD.

This study has some limitations. Firstly, the cross-sectional design of this study can not be used to determine causal relationships. Secondly, in this study, stigma was measured by a binary answers of “yes” or “no”, not by a scale, the validation would be affected to some extent. Thirdly, we gave little consideration on the relations between SI and suicidal plans and suicidal attempts, which need to be further researched in the future. The fourth limitation was that this paper lacked of a comparison sample of non-shidu parents. Finally, the impacts of other variables, such as family and community capitals [52], resilience [16] and stress [56], were not examined in the present study, these variables should be taken into account in future studies.

## Conclusion

A high prevalence of SI among shidu parents was found in our study. Our study also demonstrated that PTSD and stigma were important risk predictors of SI and social support is a protective factor of SI. Furthermore, stigma aggravated the effect of PTSD on SI while social support buffered the effect of PTSD on SI among shidu parents. This study emphasized the importance of decreasing stigma and increasing social support to reduce SI in shidu parents with PTSD.

## Data Availability

The datasets used and analysed during the current study are available from the corresponding author(Yang Wang) on reasonable request.

## Abbreviations

PTSD: Post-traumatic stress disorder
SI: Suicidal ideation
